# A preliminary study on the effects of insole material and foot strike pattern on shock attenuation during running

**DOI:** 10.3389/fspor.2026.1784118

**Published:** 2026-04-13

**Authors:** Yoshiki Horiguchi, Hiroaki Noro, Taira Yoshida, Toshio Yanagiya

**Affiliations:** 1 Graduate School of Health and Sports Science, Juntendo University, Chiba, Japan; 2 Faculty of Health and Sports Science, Juntendo University, Chiba, Japan; 3 Faculty of Sports and Health Studies, Heisei International University, Saitama, Japan; 4 Institute of Sports Sciences & Medicine, Juntendo University, Chiba, Japan

**Keywords:** Foot Strike Pattern, ground reaction (forces), insole material, running, shock attenuation, sorbothane, tibial acceleration

## Abstract

**Introduction:**

We aimed to investigate the effect of Sorbothane insole insertion and foot strike patterns on shock attenuation during running.

**Methods:**

Nine male students participated in the study. Running was performed on a 15 m runway at a constant speed of 3.33 m/s. Two types of insoles (EVA and Sorbothane) and two-foot strike patterns (forefoot and rearfoot) were used, creating four experimental conditions. The tibial acceleration and vertical ground reaction force (GRF) were measured, and their peak and loading rate were calculated. A two-way ANOVA (insole × foot strike pattern) was performed.

**Results:**

Tibial acceleration showed no significant interaction. However, the peak and loading rate of vertical GRF were significantly higher in the rearfoot strike pattern. The Sorbothane insole significantly reduced the loading rate only in the rearfoot strike condition. Ankle joint angles showed differences depending on foot strike and insole type.

**Conclusion:**

These findings suggest that the foot strike pattern may has a greater influence on impact characteristics than the difference in insole material under the present conditions. While Sorbothane insoles may offer some benefits in reducing impact loading during rearfoot strike, further studies with larger sample sizes are required to confirm these effects.

## 1 Introduction

During the first 50 milliseconds of the stance phase in running, the foot encounters a collision force approximately 1.5 to 5 times the runner's body weight (1). Such high-impact forces at foot strikes are associated with several running-related conditions, including hemolysis, tibial stress fractures, shin splints, and plantar fasciitis (1–4). The incidence of running-related injuries varies widely, ranging from 19.4% to 92.4%, with tibial stress fractures representing 1.5% to 31% of all such injuries (5). The wide range of incidence rates in running-related injuries and stress fractures may be attributed not only to differences in participant characteristics but also to inconsistencies in injury definitions and diagnostic criteria among studies (5). Running is one of the most popular forms of physical activity; however, the incidence of running-related injuries is high, with reports suggesting that approximately 20%–80% of runners experience at least one injury per year (5). These injuries can lead to interruptions in training, decreased of performance, and a increased risk of chronic conditions. Furthermore, impact forces during running are known to vary depending on factors such as foot strike pattern (6), potentially increasing the risk of injury under specific conditions.

The heel pad, composed of fatty tissue in the posterior part of the foot, serves to cushion impact transients but often falls short of sufficiently reducing impact loading to prevent injuries (7). Insufficient attenuation of impact forces may increase the transmission of mechanical loads to the musculoskeletal system, thereby elevating the risk of running-related injuries. Consequently, footwear plays a crucial role in injury prevention, particularly through the effectiveness of midsole materials such as ethylene-vinyl acetate (EVA), gels, rubber, resin, or airbags (8). Although cushioned insoles have been reported to reduce the risk of stress fractures and overuse injuries (9–11), research findings on their protective effects remain inconclusive (12–14).

Sorbothane (SRB), known for its shock-absorbing properties, is used in running shoes and various daily products to reduce shock and vibration (15). Previous studies have shown that SRB insoles effectively attenuate impact during activities such as walking, running, falling, and military training (14, 16, 17). Specifically, cushioned insoles with SRB have been shown to significantly reduce the peak vertical ground reaction force, loading rate, and tibial acceleration during running (1). Kinematic adjustments, such as reduced knee flexion at foot-ground contact, may also occur due to diminished impact forces when using cushioned insoles (1).

Moreover, different foot strike patterns (FSP) can significantly alter the magnitude and timing of collision forces on the foot during running (18). Although FSP are primarily categorized into three types, rearfoot strike (RFS), characterized by initial ground contact with the heel; midfoot strike (MFS), where the heel and forefoot contact the ground nearly simultaneously; and forefoot strike (FFS), characterized by initial contact with the forefoot — they can also be categorised by the location of initial ground contact. Rearfoot strikers experience a higher rate of musculoskeletal injuries due to specific impact transients associated with this strike pattern (19). In addition, FSP influences not only external loading parameters but also internal muscle–tendon behavior. Our previous work demonstrated that forefoot strike (FFS) running was associated with shorter fascicle length and greater tendon stretch and recoil of the gastrocnemius medialis compared with rearfoot strike (RFS) (20). These findings suggest that the mechanical behavior of the muscle–tendon unit differs substantially between FSP, possibly affecting how impact shock is internally absorbed or transmitted during foot–ground contact.

Although FFS is often considered superior to RFS because of its reduced impact forces immediately after ground contact, previous studies have reported that approximately 98% of recreational marathon runners adopt a rearfoot or midfoot strike pattern, with only 1.4% classified as forefoot strikers (21). Therefore, most recreational runners may need to attenuate impact forces at ground contact not only by modifying their foot strike pattern but also by utilizing shock-attenuating materials such as Sorbothane in their insoles or midsoles. This study aimed to explore the effects of SRB insoles and FSP on shock attenuation during running at a controlled sub-four-hour marathon pace. We hypothesized that a Sorbothane insole would attenuate impact forces to a similar extent as changing the foot strike pattern from rearfoot to forefoot strike.

## 2 Methods

### 2.1 Participants

Nine male university students who were habitual runners participated in this study. They engaged in running several times per week and had not sustained any running-related injuries in the six months prior to testing. Their mean ± SD physical characteristics were as follows: age 20.8 ± 0.67 years, height 172 ± 4.7 cm, body mass 63.1 ± 4.96 kg, and shoe size 26.0 cm. Before the experiments, all participants received verbal and written explanations of the study objectives and procedures and provided written informed consent. The study complied with the ethical standards outlined in the Declaration of Helsinki and was approved by the Research Ethics Committee of Juntendo University (approval no. JUGS-S-30-27).

### 2.2 Running trials

Participants were instructed to run at a constant speed of 3.33 m s−¹ (equivalent to 5 min·km−¹) along a 15 m indoor runway. Running speed was monitored using a laser velocity gauge (Laveg Sport LDM-300C, Jenoptik, Germany). Trials in which running speed deviated by more than ± 5% from the target were repeated to ensure consistent test conditions. All participants were habitual rearfoot strikers. Prior to data collection, sufficient practice trials were conducted to ensure that participants could consistently reproduce the instructed foot strike pattern.

### 2.3 Shoes, insole materials, and foot strike patterns

All participants wore identical running shoes (SPALDING JN-963, 26.0 cm, 285 g per shoe; Achilles Corporation, Japan). The shoes had ethylene-vinyl acetate (EVA) midsoles and plastic-rubber outsoles. Two types of insoles were examined: the original EVA insoles supplied with the shoes (42 deg hardness; ≈32 g per insole, 3 mm forefoot thickness, 4 mm rearfoot thickness), and Sorbothane (SRB) insoles designed for shock attenuation (Sorbo Super Light RO1370, Achilles Corporation, Japan; 52 deg hardness ≈165 g per insole, 5 mm forefoot thickness, 6 mm rearfoot thickness). The study evaluated the effects of insole material and foot-strike pattern (FSP) on shock attenuation under four experimental conditions:

(1) EVA/FF—forefoot strike with EVA insole,
(2) EVA/RF—rearfoot strike with EVA insole,
(3) SRB/FF—forefoot strike with SRB insole, and
(4) SRB/RF—rearfoot strike with SRB insole.

Each participant performed two successive trials under every condition, with condition order randomized across participants. The mean of the two trials was used for analysis to enhance reliability.

### 2.4 Tibial acceleration

**Figure 1:**
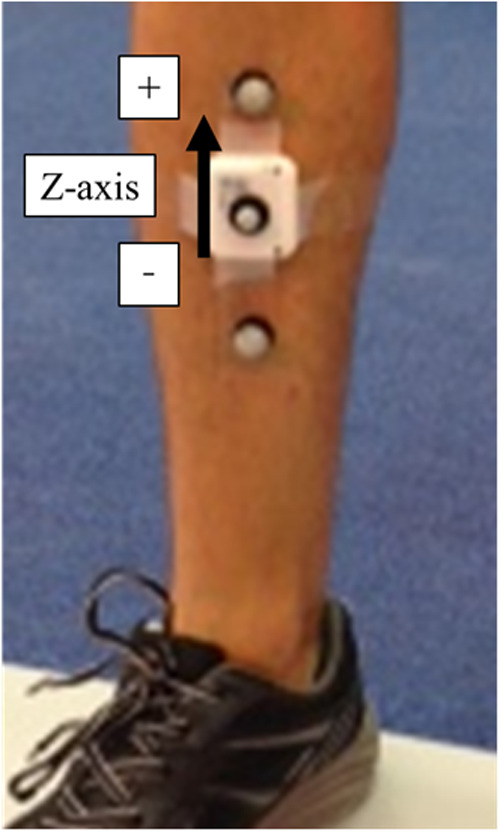
Placement of the tibial accelerometer and orientation of the vertical axis.

Tibial acceleration during the contact phase was measured as an index of impact using a wireless tri-axial accelerometer (WAA-010, Wireless Technology Inc., Japan) sampling at 1,000 Hz. The sensor's measurement range was ±16 G, and its dimensions were 0.039 × 0.044 × 0.012 m (width × height × depth), weighing 20 g. As shown in Figure 1, the accelerometer was attached to the anterior surface of the tibial midshaft of the right leg using double-sided adhesive tape and secured with elastic wrapping. The vertical axis of the accelerometer was aligned with the longitudinal axis of the tibia, with the positive direction oriented superiorly. Acceleration data were recorded in three directions (vertical z, lateral x, anterior–posterior y), but only the peak vertical (*z*-axis) value during contact was used for analysis.

### 2.5 Ground reaction forces

Ground reaction force (GRF) data in three orthogonal directions were collected at 1 kHz using two force platforms (0.9 × 0.6 m, Model 9287C/CA, Kistler, Switzerland) positioned around the 9 m point of the runway. The GRF signals were low-pass filtered at 100 Hz using a fourth-order zero-lag Butterworth filter (1) and normalized to each participant's body weight. From the vertical GRF, the impact peak (IP) and loading rate (LR) were determined following Lieberman et al. (6).

### 2.6 Kinematic measurements

Three-dimensional kinematic data were captured using an eight-camera optical motion-capture system (VICON MX, Oxford Metrics, UK) operating at 250 Hz and synchronized with the force platforms. Retroreflective markers were placed on predefined anatomical landmarks according to the Plug-in-Gait model. Marker coordinate data were low pass filtered at 9 Hz (6). Only the knee and ankle joint angles were analyzed. Knee and ankle joint angles were calculated at touchdown (KJA@TD and AJA@TD, respectively), minimum flexion (KJAmin and AJAmin, respectively), and their respective angle displacements (*Δ*KJA and *Δ*AJA, respectively).

### 2.7 Statistical analysis

All data were first tested for normality using the Shapiro–Wilk test. For normally distributed variables, a two-way repeated-measures analysis of variance (ANOVA) was performed with two within-subject factors: insole material (EVA, SRB) and foot-strike pattern (FF, RF). When a significant interaction was found, *post-hoc* comparisons were conducted with Bonferroni correction.

Results are presented as mean ± SD, and the significance level was set at *p* < 0.05. All analyses were performed using JASP (v 0.19.1, JASP Team, 2024). Effect sizes (partial *η*²) were reported, and a sensitivity analysis was conducted using G*Power (v 3.1). For a 2 × 2 within-subject ANOVA (*α* = 0.05, 1–*β* = 0.80, *ε* = 1), with *n* = 9 and assumed within-subject correlation r = 0.50, the minimum detectable effect size was f = 0.53 (partial *η*² = 0.22). Therefore, the study was adequately powered to detect medium-to-large effects but was underpowered for small effects. Observed within-subject correlations between FF and RF conditions ranged from −0.05 to 0.93 (median *r* = 0.75), indicating that the assumed *r* = 0.50 was conservative. Lower correlations for variables such as the first impact peak and ankle angles reflect inherent biomechanical differences between FF and RF rather than measurement error.

## 3 Results

All nine participants successfully completed all four experimental conditions: two insole materials (EVA and SRB) × two foot-strike patterns (FF and RF). No trials were excluded due to technical or procedural errors, and all data satisfied the normality assumption as confirmed by the Shapiro–Wilk test (*p* > 0.05). Because both within-subject factors had only two levels, sphericity and homogeneity of variance tests were not required. The two-way repeated-measures ANOVA results are summarized below for each category of variables.

Section 3.1 presents the findings for tibial acceleration, followed by 3.2 for ground-reaction-force variables and 3.3 for joint kinematics.

### 3.1 Tibial acceleration

The results for tibial acceleration are summarized in Table 1. A two-way repeated-measures ANOVA showed no significant main effects of insole type [*F*(1, 32) = 0.025, *p* = 0.876, *η*² < 0.001] or foot-strike pattern [*F*(1, 32) = 0.042, *p* = 0.839, *η*² = 0.001], and no interaction [*F*(1, 32) = 1.315, *p* = 0.260, *η*² = 0.039]. *post-hoc* analyses revealed no significant pairwise differences (all *p* > 0.05).

**Table 1:** Tibial acceleration during foot contact of running in various foot strike patterns.

	EVA/FF	SRB/FF	EVA/RF	SRB/RF	Insole	FSP	Int
Tibial acceleration [G]	8.77 (2.39)	9.67 (2.63)	9.99 (2.72)	8.82 (3.04)	ns	ns	ns

Mean (±standard deviation).

EVA, ethylene-vinyl acetate; FF, forefoot strike; SRB, sorbothane; RF, reaction force; FSP, foot strike pattern; Int, interaction; ns, not significant.

### 3.2 Ground reaction forces

The results for ground reaction forces are summarized in Table 2.

**Table 2:** Vertical ground reaction data in each condition.

	EVA/FF	SRB/FF	EVA/RF	SRB/RF	Insole	FSP	Int
Impact Peak [BW]	1.52 (0.211)	1.51 (0.240)	2.02 (0.202)	1.91 (0.255)	ns	*P* < 0.001	ns
Loading Rate [BW/s]	50.38 (7.90)	48.94 (10.00)	66.03 (13.810)	59.71 (16.90)	ns	ns	*P* < 0.01
Fmax [BW]	3.02 (0.277)	2.97 (0.252)	2.04 (0.282)	1.91 (0.317)	ns	*p* < 0.001	ns

Mean (±standard deviation).

EVA, ethylene-vinyl acetate; FF, forefoot strike; RF, rearfoot strike; FSP, foot strike pattern.

Int, interaction; ns, not significant; IP, impact peak; LR, loading rate; Fmax, maximum value of vertical ground reaction; SRB, sorbothane.

IP: EVA/RF vs. EVA/FF (*p* = 0.00267), SRB/FF vs. EVA/RF (*p* = 0.0001), SRB/RF vs. EVA/FF (*p* = 0.02557), SRB/RF vs. SRB/FF (*p* < 0.00001).

LR: SRB/RF vs. EVA/RF (*p* = 0.03475).

Fmax: EVA/RF vs. EVA/FF (*p* < 0.00001), SRB/FF vs. EVA/RF (*p* < 0.00001), SRB/RF vs. EVA/FF (*p* < 0.00001), SRB/RF vs. SRB/FF (*p* < 0.00001).

#### 3.2.1 Maximum force (fmax)

A significant main effect of foot-strike pattern was observed [*F* (1, 32) = 8.105, *p* = 0.008, *η*² = 0.201], with forefoot strike (FF) producing greater Fmax than rearfoot strike (RF). No significant main effect of insole [*F*(1, 32) = 0.191, *p* = 0.665, *η*² = 0.005] or interaction [*F* (1, 32) = 1.753 × 10−⁴, *p* = 0.990, *η*² < 0.001] was found.

Descriptive statistics showed higher Fmax in FF than RF for both insoles (EVA: 3.001 ± 0.292 vs. 2.763 ± 0.199; SRB: 2.966 ± 0.252 vs. 2.726 ± 0.254). Although the main effect of foot-strike pattern was significant (*p* = 0.008, *η*² = 0.201), *post-hoc* pairwise comparisons did not reach significance (all *p* > 0.10).

#### 3.2.2 Impact peak (IP)

Homogeneity of variance was confirmed [*F* (3, 32) = 0.083, *p* = 0.969]. A significant interaction was observed between insole and pattern [*F*(1, 32) = 23.897, *p* < 0.001, *η*² = 0.422], whereas the main effects of insole [*F*(1, 32) = 0.422, *p* = 0.520, *η*² = 0.007] and pattern [*F*(1, 32) = 0.349, *p* = 0.559, *η*² = 0.006] were not significant. For the EVA insole, the impact peak was greater in RF than FF (2.020 ± 0.278 vs. 1.517 ± 0.244), while for the SRB insole, the reverse trend was observed (FF > RF; 1.906 ± 0.317 vs. 1.511 ± 0.258). *post-hoc* analysis revealed that the impact peak was significantly greater in RF than FF for EVA (*p* = 0.003, *d* = 1.83) and greater in FF than RF for SRB (*p* = 0.023, *d* = 1.43). Additionally, SRB–FF exceeded EVA–FF (*p* = 0.026, *d* = 1.41), and EVA–RF exceeded SRB–RF (*p* = 0.002, *d* = 1.85).

#### 3.2.3 Loading rate

A significant main effect of foot-strike pattern was found [*F* (1, 32) = 9.810, *p* = 0.004, *η*² = 0.228], with RF showing higher loading rates than FF. No significant main effect of insole [*F* (1, 32) = 0.853, *p* = 0.363, *η*² = 0.020] or interaction [*F* (1, 32) = 0.332, *p* = 0.569, *η*² = 0.008] was observed. Descriptive statistics indicated higher loading rates in RF than FF for both insoles (EVA: 66.03 ± 13.41 vs. 50.40 ± 9.07; SRB: 59.71 ± 16.68 vs. 48.93 ± 9.99). *post-hoc* results showed a marginal difference between EVA–FF and EVA–RF (*p* = 0.061, *d* = 1.24) and a significant difference between SRB–FF and EVA–RF (*p* = 0.035, *d* = 1.35).

### 3.3 Kinematics

**Figure 2:**
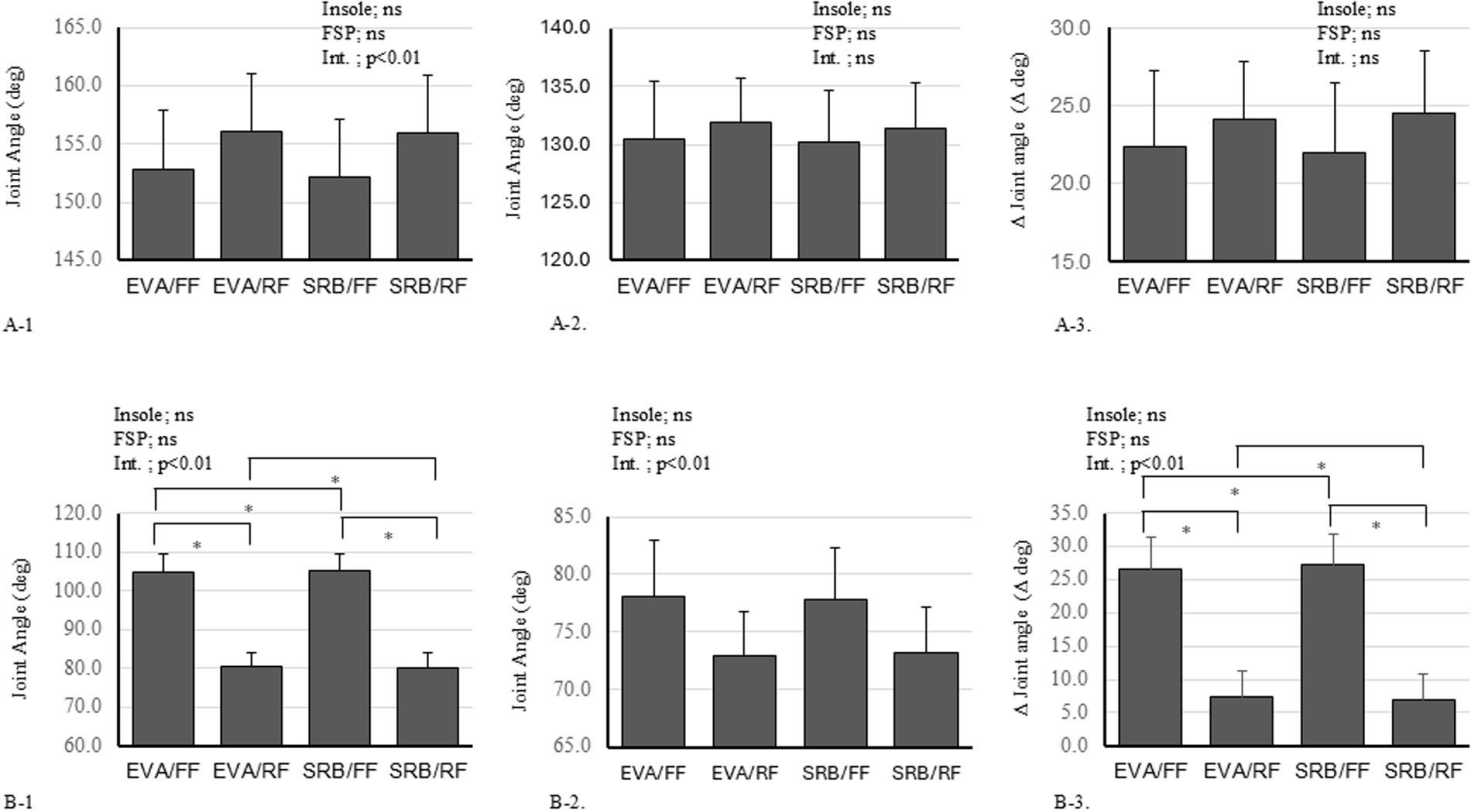
Joint angle differences related to insole material and foot strike pattern in the lower limb. **(A-1)** Knee joint angle at touchdown, **(A-2)** minimum knee joint angle, **(A-3)** *Δ* knee joint angle, **(B-1**) ankle joint angle at touchdown, **(B-2)** minimum ankle joint angle, and **(B-3)** *Δ* ankle joint angle. * indicates a significant difference (*p* < 0.05). EVA, ethylene-vinyl acetate; FF, forefoot strike; RF, rearfoot strike; SRB, sorbothane; FSP, foot strike pattern; ns, not significant.

The results for lower extremity joint angles are summarized in Figure 2.

#### 3.3.1 Ankle joint

Ankle angle at touchdown (Ankle JA@TD):

A significant main effect of foot-strike pattern was found [*F* (1, 32) = 190.729, *p* < 0.001, *η*² = 0.856], with FF showing a more plantarflexed angle at touchdown (mean difference = 24.61°, *p* < 0.001). Neither insole [*F* (1, 32) = 0.003, *p* = 0.958] nor interaction [*F* (1, 32) = 0.049, *p* = 0.826] was significant.

Ankle minimum angle (Ankle Min JA):

A significant main effect of foot-strike pattern was observed [*F* (1, 32) = 9.896, *p* = 0.004, *η*² = 0.236], with FF remaining more plantarflexed than RF (mean difference = 4.89°, *p* = 0.004). No effects of insole or interaction were found (both *p* > 0.85).

Ankle range of motion (Ankle *Δ*JA):

Foot-strike pattern significantly affected the range of motion [*F* (1, 32) = 229.822, *p* < 0.001, *η*² = 0.877], with FF exhibiting a larger excursion than RF (mean difference = 19.71°, *p* < 0.001). Neither insole (*p* = 0.949) nor interaction (*p* = 0.626) reached significance. The low within-subject correlation for this variable (*r* = 0.36) similarly represents the expected divergence in movement patterns between strike types rather than measurement variability.

#### 3.3.2 Knee joint

No significant main effects or interactions were found for knee joint angles.

For the minimum knee angle, neither insole [*F*(1, 32) = 0.113, *p* = 0.739, *η*² = 0.003], pattern [*F*(1, 32) = 0.798, *p* = 0.378, *η*² = 0.024], nor interaction [*F*(1, 32) = 0.007, *p* = 0.936] was significant. Similarly, for the range of knee motion (Knee *Δ*JA), no significant effects were found for insole [*F* (1, 32) = 0.002, *p* = 0.962], pattern [*F* (1, 32) = 2.511, *p* = 0.123], or interaction [*F* (1, 32) = 0.080, *p* = 0.779].

## 4 Discussion

This study aimed to clarify the effects of foot strike patterns and insole materials (Sorbothane vs. EVA) on tibial acceleration, ground reaction forces, and running kinematics at a sub-four marathon pace, focusing on impact forces as a contributing factor to running-related injuries among recreational runners. This study has practical significance because this marathon pace corresponds to the running speed of many recreational runners, among whom running-related injuries are frequently reported.

As a result, although neither foot-strike pattern nor insole material significantly affected tibial acceleration, differences between conditions were observed in several ground reaction force indices and ankle kinematics. Ground reaction force characteristics were primarily modulated by foot-strike pattern, whereas the influence of insole material was limited and condition-specific. In this section, these findings are discussed from measurement and biomechanical perspectives, with reference to previous studies.

As shown in Table 1, no significant differences in tibial acceleration were observed, regardless of differences in foot strike patterns or insole materials in this study. This suggests that an accelerometer attached to the skin may not be sufficiently sensitive to detect high-frequency impacts at initial ground contact, rather than indicating that impact forces did not differ between conditions. While a high correlation (r ≈ 0.8) between tibial acceleration and impact force has been reported (22), other studies have indicated that significant differences are difficult to detect under subtle condition changes (23). Additionally, several studies have reported no significant differences in the tibial acceleration across different running surfaces (24–26).

Tibial acceleration is thought of as a secondary index reflecting the vibrational response of soft tissue rather than a direct measure of impact force from ground reaction (27). Therefore, it is not appropriate to conclude that impact forces did not differ between the conditions based on solely on the absence of significant differences in tibial acceleration. The results of the present study suggest that when evaluating impact, it is necessary to use multiple indices, including not only tibial acceleration but also ground reaction force and plantar pressure.

As shown in Table 2, analysis of ground reaction forces revealed that impact peak (IP) and loading rate (LR) differed significantly according to foot strike pattern. These indices are associated with the risk of running-related injuries (6), and the present study also showed higher value for rearfoot strike than forefoot strike. This result can be attributed to the fact that rearfoot produces a double peak force waveform due to initial heel impact, whereas forefoot strike produces a unimodal waveform with attenuated impact.

Although forefoot strike running is an effective as the strategy for decreasing the impact of foot ground contact, it is known to increase the eccentric load on the triceps surae muscles and the Achilles tendon (28). Therefore, while changing foot strike patterns are effectively reduce impact, there is a trade-off in terms of increased musculotendinous loading and accumulated fatigue, which may elevate the risk of running-related injuries.

In the present study Sorbothane insoles were used, however it is appropriate to interpret the results from the broader perspective of shock-attenuating materials in general, rather than limiting the discussion to one specific material. Previous studies have indicated that the hardness and viscoelasticity of midsole materials significantly affect shock absorption during running. Moreover, shock absorption mechanisms differ according to foot strike pattern.

According to Clarke et al. the rate of development of vertical ground reaction force decreases with an increase in shoe cushioning (29). Nigg et al. indicated that impact acceleration increases in accordance with midsole hardness (30). Law et al. (31), meanwhile, reported an increased vertical average loading rate and decreased ground contact time in accordance with decreased midsole thickness. Furthermore, Yang et al. reported that the shock duration was prolonged because of decreased vertical ground reaction force (VALR) due to the combination of a softer midsole and a softer ground surface (32). These studies indicated that the hardness and thickness of the midsole directly affect both the magnitude and duration of loading during foot strike.

Moreover, shock absorbing system differs by the foot strike pattern. Lieberman et al. suggested that, during forefoot strike running, the impact peak immediately after foot–ground contact is attenuated and shock absorption is dynamically regulated by the muscle–tendon system (6). Therefore, the effect of shock-attenuating materials should be understood in relation to the body's intrinsic shock absorption mechanisms, which differ depending on foot strike patterns.

In this study, a tendency towards a smaller impact was observed with the Sorbothane insole compared to it in the EVA insole, although no significant main effect was observed in the IP and LR. These results suggest that viscoelastic materials have the potential to disperse impact at foot–ground contact. The differences in insole materials may induce differences in foot contact time and running kinematics in the lower limb joints due to their viscoelastic properties. In fact, there are significant differences in the joint kinematics of the ankle joint at touchdown and the delta joint angle in both the forefoot and rearfoot conditions between EVA and SRB. However, the slightly larger delta joint angle in SRB compared to EVA may decrease running performance due to the longer contact time, even though it prevents larger impacts during contact. Therefore, these results indicate that the effectiveness of shock-attenuating materials depends not only on the materials themselves, but also on a combination of kinematic factors, such as foot strike pattern and contact time.

As shown in Figure 2, analysis of kinematic variables revealed significant differences between foot strike patterns, whereas no effect of insole material was observed. Therefore, we can conclude that the selection of foot strike pattern is more effective in attenuating impact than the selection of insole material. This is especially the case given the limited range of materials selected for this study.

Analysis of ankle joint angles indicated that participants successfully reproduced the instructed foot strike patterns. Therefore, the observed differences in ground reaction force can be attributed to changes in running kinematics rather than to insole materials.

Several limitations of the present study should be acknowledged. First, tibial acceleration was measured using a skin-mounted accelerometer, which may be influenced by soft tissue artifacts and may not fully reflect underlying bone acceleration. Second, the relatively small sample size (*n* = 9) limited the statistical power to detect small effects. Based on the design sensitivity of the present study (detectable partial *η*² = 0.22 at 80% power), the absence of significant effects—particularly for tibial acceleration and knee kinematics—should not be interpreted as evidence of no effect, but rather as an indication that any potential differences were likely small. In addition, several variables exhibited discrepancies between omnibus ANOVA results and *post-hoc* pairwise comparisons, which may reflect the conservative nature of *post-hoc* corrections combined with limited statistical power. Low within-subject correlations observed for variables such as the first vertical peak and ankle joint angles are likely attributable to the fundamentally distinct mechanical characteristics of forefoot and rearfoot strike patterns, rather than insufficient measurement reliability. Therefore, caution is warranted when interpreting null findings or non-significant pairwise differences, and further studies with larger sample sizes and alternative measurement approaches are required to clarify the influence of insole materials under different foot-strike conditions. Third, only male participants were included to minimize the potential influence of sex-related differences in running biomechanics, which may limit the generalizability of the findings.

Furthermore, the present study did not examine the viscoelastic characteristics or response time of the shock-attenuating materials in detail. Future studies should evaluate the relationship between material characteristics and foot strike pattern and running velocity, considering these factors simultaneously.

These results suggest that tibial acceleration is limited in its ability to reflect and measure the shock magnitude. Conversely, ground reaction force accurately reflects the effect of foot strike pattern and shock-attenuating materials. This study suggests that viscoelastic materials in the rear foot strike running provide a shock attenuation effect, which could inform the design of footwear for shock reduction and improved running form.

## 5 Conclusion

Foot strike pattern significantly influenced ground reaction force variables, whereas no significant effects were observed for tibial acceleration or insole material. Although the Sorbothane insole showed a tendency to reduce impact loading during rearfoot strike, this effect was not statistically significant. Therefore, the null hypothesis regarding the effect of insole material was not rejected. Further studies with larger sample sizes are required to confirm these findings.

## Data Availability

The original contributions presented in the study are included in the article/Supplementary Material, further inquiries can be directed to the corresponding author/s.
